# Apperceptive and Associative Forms of Phonagnosia

**DOI:** 10.1007/s11910-023-01271-5

**Published:** 2023-05-03

**Authors:** Guido Gainotti, Davide Quaranta, Simona Luzzi

**Affiliations:** 1 Institute of Neurology, Catholic University of the Sacred Heart, Largo A. Gemell, 8, 00168 Rome, Italy; 2 Neurology Unit, Department of Science of Elderly, Neuroscience, Head and Neck and Orthopaedics, Fondazione Policlinico A. Gemelli, IRCCS, Rome, Italy; 3 Department of Experimental and Clinical Medicine, Polytechnic University of Marche, Ancona, Italy

**Keywords:** Apperceptive and associative phonagnosia, Voice discrimination, Voice familiarity feelings, Core TVAs, Voice extended system

## Abstract

**Purpose of Review:**

Pronagnosia is a rare acquired or developmental pathological condition that consists of a selective difficulty to recognize familiar people by their voices. It can be distinguished into two different categories: apperceptive phonagnosia, which denotes a purely perceptual form of voice recognition disorder; and associative phonagnosia, in which patients have no perceptual defects, but cannot evaluate if the voice of a known person is or not familiar. The neural substrate of these two forms of voice recognition is still controversial, but it could concern different components of the core temporal voice areas and of extratemporal voice processing areas. This article reviews recent research on the neuropsychological and anatomo-clinical aspects of this condition.

**Recent Findings:**

Data obtained in group studies or single case reports of phonagnosic patients suggest that apperceptive phonagnosia might be due to disruption of the core temporal voice areas, bilaterally located in the posterior parts of the superior temporal gyrus, whereas associative phonagnosia might result from impaired access to structures where voice representations are stored, due to a disconnection of these areas from structures of the voice extended system.

**Summary:**

Although these results must be confirmed by further investigations, they represent an important step toward understanding the nature and neural substrate of apperceptive and associative forms of phonagnosia.

## Introduction

The term “*phonagnosia*” (from the ancient Greek “Phon” = “voice” and “agnosia” = “not knowing”) designates a rare pathological condition, which consists of a more or less selective disorder of familiar voice recognition. It was proposed by Van Lancker and Canter [1], in analogy with the term “*prosopagnosia*” (“prósōpon” = face”) previously coined by Bodamer [2] to designate the inability to recognize the face of known people.

The similarity between these two words and the earlier formulation of the term “prosopagnosia” with respect to the term “phonagnosia” rightly suggests that both a parallelism and a prevalence of face over voice may characterize these two kinds of people recognition disorders.

The parallelism stems from the fact that both face and voice play a critical role in the non-verbal identification of known people, due to a sequence of similar processing mechanisms, which include: (a) lower-level perceptual processes, leading to the formation of modality-specific representations; (b) a locus of convergence of the output of these processes, resulting in the construction of multimodal semantic representations, allowing to access the corresponding proper names. Framed in terms of current neuroscientific models of person recognition [3–9], these processing mechanisms develop within two interconnected systems: a core system, which processes modality-specific perceptual and identity information of the face or voice, and an extended system, which encodes multi-modal semantic information about a person, such as the occupation or the name. The disruption of each of these processing steps can be diagnosed with specific tools, allowing to classify the (face or voice) recognition disorder into different diagnostic categories. The term “*apperceptive*” denotes a purely perceptual disorder, whereas the term “*associative*” indicates a disruption of the corresponding modality-specific representations, and the term “*multimodal*” denotes a disruption of the representations arising from the integration of different person recognition modalities.

The parallelism between face and voice recognition mechanisms is reinforced by the non-verbal nature of these person identification modalities, which leads to a prevalent right hemispheric lateralization of mechanisms underlying face and voice recognition (e.g., [6, 7, 10–12].

A further parallelism between prosopagnosia and phonagnosia stems from the distinction between acquired and congenital forms of people recognition disorders because, in recent times, the study of face and voice recognition disorders resulting from acquired brain diseases has been accompanied by an increasing interest for congenital forms of these disorders.

In contrast with these similarities, which have inspired to Belin et al. [5] and Yovel and Belin [13] the notion that voice may be considered as an “auditory face,” there are, however, important behavioral and neuro-anatomical differences between these two modalities of person identification. An important behavioral difference, documented by several authors (e.g., [14, 15]), resides in the greater difficulty met in person recognition through voice in comparison to face. Bredart et al. [16] have, indeed, shown that this difference is not simply due to the fact that in the media we see celebrities’ faces more frequently than we hear their voices, because a similar face advantage can be observed using the faces and voices of personally known persons.

More neurobiologically grounded explanations could reside in the lower degree of functional specificity shown by areas involved in voice perception in comparison to those involved in face perception [9, 17] or in the different temporal dimensions of face and voice processing, given that faces can be discerned from a single snapshot [18], whereas voices constitute dynamic stimuli for which receivers must integrate information over time. In any case, the greater difficulty met in person identification through voice rather than through face can explain the relative rarity of group studies or single case reports dealing with voice (in comparison to face) recognition disorders. The low number of these investigations could, in turn, at least in part, explain the disagreement still existing about the selectivity, the neural correlates, and the lateralization of the different forms of voice recognition disorders. The aim of the present synthetic review will, therefore, consist in presenting and discussing some recent data on this subject.

## Acquired Forms of Apperceptive and Associative Phonagnosia

The expression “*apperceptive phonagnosia*” denotes a purely perceptual form of voice recognition disorder and is used when the subject cannot evaluate if sentences spoken by unfamiliar persons are pronounced by the same or by different speakers. It is generally assumed that the neural substrate of this lower-level voice recognition disorder may be a lesion encroaching upon the temporal voice areas (TVAs), located bilaterally along the upper banks of the superior temporal gyri and sulci [19, 20].

The expression “*associative phonagnosia*” is instead used to indicate the disruption of the voice representations built by these perceptual mechanisms and is characterized by the inability to evaluate if the voice of a known person is or not familiar. This defect is due to the fact that familiarity feelings are automatically generated when the incoming voice is matched with a previously established voice-reference pattern. The absence of familiarity feelings points, therefore, to a disruption of the corresponding voice representations. The neural substrate of this level of voice recognition is, however, still controversial, because it could concern components of the core TVAs and aspects of the extratemporal voice processing areas.

Both group studies of voice recognition disorders and neuropsychological investigations of subjects showing selective forms of phonagnosia have tried to clarify these issues. The first group studies were conducted by Van Lanker et al. [21, 22] and showed that behavioral and neuroanatomical dissociations can be found between impairments of voice discrimination and of voice identification, because the former are often associated with temporal lobe damage of either hemisphere, whereas the latter are accompanied by damage to inferior and lateral parietal regions of the right hemisphere.

These data, however, have been only in part confirmed by investigations conducted on this subject in more recent times. Thus, in a very well-controlled group study of phonagnosia in patients with unilateral focal brain lesions, Roswandowitz et al. [23•] showed that the right posterior/mid-temporal lobe is critical for recognizing newly learned unfamiliar, in comparison to familiar, voices. This led Roswandowitz et al. [23•] to suppose that right posterior/mid-temporal lobe structures may support the acoustical voice-identity feature analysis necessary to establish a new voice-specific reference pattern. In another group study of patients with unilateral temporal lobe tumors, Papagno et al. [24], using standardized tests of unknown voice discrimination and of famous voice recognition [25], showed that voice discrimination disorders are due to lesions involving the whole right anterior temporal lobe and extending to lateral portions of the temporal and frontal lobes. These authors also made two other observations, as they showed: (a) that some patients with a right temporal tumor had a normal performance in famous voice identification, in spite of having severe voice discrimination disturbances; and (b) that familiarity judgments (testified by an increased number of false alarms) are impaired by lesions restricted to the right anterior temporal lobe (ATL). The relationship between the increased number of false alarms (FA) in voice familiarity tasks and damage to the right ATL was confirmed by Papagno et al. [26] and Piccininni et al. [27•] in further studies conducted on patients with neoplastic or degenerative lesions and in normal participants tested after anodal transcranial direct current stimulation (tCDS), over the left or right ATL. This association of voice discrimination errors and FA was considered as due to the greater complexity of voice processing that could impact of the capacity to form stable and well-structured representations, allowing to evaluate if a presented voice matches or not with an already known voice.

Only in part consistent with results obtained by group studies of voice recognition disorders are the data obtained in single case studies of selective forms of phonagnosia, reported by Hailstone et al. [28], Luzzi et al. [29••], and Didic et al. [30]. It is interesting to note that in all these patients lesions involved the ATLs, which are considered as the locus of convergence of the perceptual inputs that lead to the construction of multimodal semantic information [31–33].

Hailstone et al. [28] reported two cases of “progressive associative phonagnosia”: QR, a 61-year-old right-handed woman, diagnosed as a’ behavioral variant frontotemporal dementia, and KL, a 72-year-old left-handed man, diagnosed as a “temporal variant frontotemporal lobar degeneration” with progressive right temporal lobe atrophy.

QR exhibited severe impairments of voice identification and familiarity judgments, with relatively preserved recognition of environmental sounds, but mildly impaired recognition of musical instruments. Her brain MRI showed bilateral fronto-temporal atrophy somewhat accentuated in the right anterior temporal lobe but extending posteriorly within the temporal lobe and including the superior temporal sulcus. On the contrary, KL showed a very mild form of phonagnosia (he found it increasingly difficult to understand unfamiliar accents) and was mainly impaired on recognition of famous faces. His brain MRI showed bilateral predominantly anterior temporal lobe atrophy, more marked on the right side and in the inferior temporal cortices, including the fusiform gyrus.

Luzzi et al. [29••] reported the case of a 48-year-old man (MM) who, after an ischemic stroke in the right hemisphere, noticed that he was unable to recognize the voice of his favorite singers. A neuropsychological examination revealed a selective impairment in famous voice recognition, with very low scores both in the familiarity evaluation and in the semantic questionnaire. These defects of voice identification contrasted with performances in the upper part of the normal range in tasks exploring voice and face discrimination and in famous face recognition. Furthermore, his results were at the ceiling in recognition of emotional prosody and of non-musical sounds and in perception and recognition of musical characteristics. Neuroimaging, carried out by means of PET and MRI, revealed two small ischemic lesions in the right lenticular and caudate nuclei and in the right temporal pole, extending posteriorly in the middle temporal gyrus.

Didic et al. [30] reported a former telephone operator (PM) with a genetic form of fronto-temporal degeneration (FTD related to a C9orf72 expansion), who, at the age of 72, began to complain of difficulty recognizing his interlocutors on the phone, but not when he met them face to face. Given his developed skill in recognizing speakers on the phone, this difficulty became a matter of concern for his family. His neurological examination and his cognitive functions were intact, and a pure tone audiogram and auditory potentials were within normal limits. However, when asked to match the voices of individuals that he knew personally with their photographs, he was able to match correctly only two voices out of ten and showed a severe impairment in retrieving semantic information about celebrities from their voice. These defects contrasted with the intact knowledge shown when he was presented with the faces and names of the same persons. The PM was, however, mildly impaired in judging whether famous voices were familiar or unfamiliar and obtained scores significantly worse than controls on a task exploiring voice discrimination. MRI showed atrophy within fronto-temporal regions, with a bilateral widening of the superior temporal sulcus, whereas PET revealed hypometabolism within the right fronto-temporal region.

## Developmental Forms of Phonagnosia

The first case of development phonagnosia was reported by Garrido et al. [34], who described the case of a 60-year-old active professional woman (KH), who maintained that she had always experienced severe voice recognition difficulties. KH was tested on behavioral tasks measuring voice and face identification, recognition of vocal emotions, speech perception, and processing of environmental sounds and music. She was impaired on tasks requiring the recognition of famous voices and the learning and recognition of new voices but not on the other tasks. With respect to the voice abilities, there was no clear evidence for a dissociation in performance between the tasks requiring memory for voices and those requiring perceptual discrimination of two voices.

Two further cases of developmental phonagnosia (AS and SP) were reported by Roswandowitz et al. [35] who identified them within more than 1000 data sets, collected from self-selected German individuals by using a web-based screening test designed to assess their voice-recognition abilities. AS was a 32-year-old female, and SP was a 32-year-old male; both were otherwise healthy and educated, had normal hearing, and showed no pathological abnormalities in brain structures. The two cases had comparable patterns of impairments, because both performed at least 2 SDs below the level of matched controls on tests that required: learning new voices, judging the familiarity of famous voices, and discriminating pitch differences between voices. However, AS performed much worse than controls on a task of voice discrimination, whereas SP’s performance was within the normal range. In a further study, Roswandowitz et al. [36••] used functional magnetic resonance imaging (fMRI) experiments to investigate the brain functional correlates of these behaviorally well-characterized forms of phonagnosia. They found that in apperceptive phonagnosic subject AS, the right-hemispheric auditory voice-sensitive regions showed lower responses than matched controls for vocal versus non-vocal sounds and for speaker versus speech recognition. On the other hand, in associative phonagnosic subject SP, the connectivity between voice-sensitive (i.e., right posterior temporal sulcus and middle/inferior temporal gyrus) and supramodal (i.e., amygdala) regions was reduced in comparison to matched controls during speaker versus speech recognition. They therefore showed that apperceptive phonagnosia is associated with the dysfunction of voice-sensitive regions, whereas in associative phonagnosia these structures are intact, but an impaired connectivity can be found between voice-sensitive and supramodal person recognition regions.

Xu et al. [37•] reported the case of AN, a 20-year-old female, with no history of neurological events or detectable lesions, who was markedly poorer than controls at identifying her most familiar celebrity voices though being normal at face recognition and in discriminating which of two speakers uttered a particular sentence. Since AN reported that she had difficulty in imagining the voices of highly familiar celebrities, this aspect of her behavior was systematically investigated by asking AN and two other phonagnosic subjects to rate the imagery of voice and non-voice stimuli. All these phonagnosic subjects gave markedly lower ratings to the imagery of voices than to those of non-voice stimuli.

## Data Supporting and Unsettling the Distinction Between Apperceptive and Associative Forms of Phonagnosia and Multimodal People Recognition Disorders

The above-mentioned results of investigations that have studied acquired and developmental forms of voice recognition disorders had substantially supported the distinction between apperceptive and associative forms of phonagnosia and multimodal people recognition disorders, but had also raised theoretical, diagnostic, and neuroanatomical problems.

From the theoretical point of view, the distinction between apperceptive and associative forms of phonagnosia is based on a hierarchical model of voice processing whereby acoustic voice processing leads to the construction of voice reference patterns (voice representations) necessary for voice identity recognition. This hierarchical model has been recently confirmed by Bestelmeyer and Muhl [17] but it contrasts with clinical observations made by Liu et al. [38] and by Papagno et al. [24] of patients with right temporal tumors who obtained a normal performance in famous voice recognition and identification, in spite of having severe voice discrimination disturbances.

From the diagnostic point of view, some results raised objections to the diagnostic criterion assuming that familiarity scores obtained in front of known (and unknown) voices may allow to identify the associative forms of phonagnosia. This judgment is, in fact, based on the number of both “hits” and that of “false alarms” (FA), but Piccininni et al. [27•] and Terruzzi et al. [39] have argued that on voice familiarity judgments, perceptual abilities impact more on FA than on hits, because the latter require a correct matching between a percept and a stable representation, whereas the former results by a wrong matching between a percept and a non-existing representation. Scores obtained on a voice familiarity judgment could, therefore, be based on both perceptual and representational components. A better diagnostic criterion could, perhaps, consist in assuming that a number of voice discrimination errors and of FA relatively higher than the number of hits may be indicative of a mainly perceptual defect, whereas a number of voice discrimination errors and of FA clearly lower than the number of hits should be indicative of a representational defect.

Finally, from the neuroanatomical point of view, some cautions about the distinction between “associative phonagnosia” and multimodal people recognition disorders were suggested by the fact that all the case reports of patients with acquired forms of associative phonagnosia in which anatomical data were available (namely patients KL and QR of Hailstone et al. [28], patient MM of Luzzi et al. [29••]) and patient PM of Didic et al. [30] were affected by lesions involving the ATLs. The anterior parts of the temporal lobes are, in fact, considered as regions of convergence of perceptual modalities, leading to the construction of multimodal people representation, rather than to that of modality-specific voice representations.

Only one of these patients, however (namely patient MM [29••]), satisfied all the criteria of a modality-specific disorder selectively affecting voice recognition requested for a diagnosis of pure associative phonagnosia. His voice recognition disorder was, in fact, modality specific, because no defect was found in face discrimination and face identification and was selective, because voice discrimination was intact. Furthermore, his results were at the ceiling in all the auditory cognitive tasks of recognition of non-verbal sounds and of emotional prosody, and in perception and recognition of musical characteristics. On the contrary, mild defects of auditory perception or of voice discrimination were found in patients QR and PM and a multimodal defect of people recognition disorder was present in patient KL. This patient showed, in fact, a mild form of phonagnosia and a severe face recognition disorder, consistent with the localization of his anterior temporal lobe atrophy, which was more marked on the right side and in the inferior temporal cortices including the fusiform gyrus.

On the other hand, behavioral and neuroimaging data suggested the presence of both perceptual and representational defects in patients PM and QR, because recognition of unfamiliar voices and of environmental sounds was significantly worse than controls in patient PM and voice discrimination was not tested (but recognition of musical instruments was impaired) in patient QR. Furthermore, in these patients, the presence of apperceptive components was also suggested by their neuroimaging data, since their brain MRI showed a fronto-temporal atrophy with the bilateral widening of the superior temporal sulcus.

A combination of apperceptive and associative components of phonagnosia was also found in patients reported for developmental forms of phonagnosia. Patients KH [34] and AS [35] showed no clear evidence for a dissociation in performance between tasks of voice identification and those of voice discrimination, whereas patients SP [35] and AN [36••] presented a more selective defect of voice identity recognition. The associative nature of phonagnosia shown by patient SP was documented by results of the fMRI experiments which showed that in this patient auditory voice-sensitive regions (i.e., right posterior middle/inferior temporal gyrus) were intact, whereas the connectivity between these regions and supramodal (i.e., amygdala) regions was reduced in comparison to matched controls during a voice identification task. On the other hand, the representational nature of phonagnosia shown by patient AN was documented by the fact that she was unable to imagine the voices (but not faces) of highly familiar celebrities. Furthermore, a similar tendency to obtain lower ratings to the imagery of voices than to the that of non-voice stimuli was also observed by Xu et al. in two other subjects with developmental phonagnosia [31–33].

## Lesion Laterality in Patients with Acquired Forms of Apperceptive and Associative

### Phonagnosia

In their pioneering studies of phonagnosia, Van Lanker et al. [21, 22] suggested that impairments of voice discrimination may be due to temporal lobe damage in either hemisphere, whereas disturbances of voice recognition may be accompanied by damage to the inferior and lateral parietal regions of the right hemisphere. However, subsequent group studies of voice recognition disorders in patients with focal unilateral brain lesions [23•, 24, 27•] and results of brain imaging studies which have investigated the cortical regions involved in distinct aspects of voice processing [36••, 37•, 38, 40, 41] have documented a dominance of the right temporal lobe, and in particular of the right posterior temporal sulcus, in the perceptual aspects of voice processing. More controversial data have been, on the contrary, obtained studying the lateralization of lesions subserving the associative forms of phonagnosia. Group studies of patients with unilateral focal brain lesions [24, 27•] have, indeed, shown that the relations between voice familiarity defects and right temporal lobe lesions are mainly due to the high number of FA in these patients, but we have said in previous sections of this review that FA can be due to disruption of both representational and perceptual components. Furthermore, Piccininni et al. [27•] have shown that their patients with right ATL lesions were significantly more impaired than those with left ATL lesions on voice familiarity judgments, but not on the retrieval of semantic information from familiar voices. More consistent right lateralization of lesions was observed in single case studies of patients with selective forms of phonagnosia [28, 29••, 30]. In patient MM, who showed the most clear-cut form of associative phonagnosia, the vascular lesion was restricted to the right temporal lobe and to the right subcortical structures, and in patients QR and PM, who exhibited a greater impairment in voice identification and familiarity judgments than in recognition of environmental sounds, the atrophy was bilateral, but more accentuated in the right temporal lobe.

## Concluding Remarks

The distinction between apperceptive and associative forms of voice recognition disorders seems proven by the existence of single cases of acquired (e.g., patient MM [29••]) or developmental (e.g., patient SP [35]) phonagnosia, showing a severe defect in familiar voice recognition in the absence of disorders in unfamiliar voice discrimination. Also well proven is the relation between apperceptive phonagnosia and disruption of the temporal voice areas (TVAs), and in particular of the right superior temporal sulci and gyri, whereas less documented is the hypothesis that disruption of (or impaired access to) voice representations may be due to a disconnection of the TVAs from structures of the voice extended system. The strongest evidence supporting this model is the result of fMRI experiments [36••], which have shown that in developmental phonagnosic patient SP the connectivity between voice-sensitive and supramodal (i.e., amygdala) regions was reduced in comparison to matched controls during a specific task of voice recognition. This result raises the problem of the role that the amygdala could play in the generation of material-specific familiarity feelings. Previous studies (e.g. [4, 42]) have considered the amygdala as part of the extended system dedicated to recognizing emotional facial expression, but Kafkas et al. [43] have shown that the amygdala can also generate familiarity feelings specific to faces in comparison to other visual stimuli (objects and outdoor scenes). To our knowledge, however, no investigation has assessed this material specificity for voices within the auditory modality. We therefore think that further studies should try to confirm in subjects with acquired or developmental forms of associative phonagnosia the results obtained by Rosandowitz et al. [36••] in patient SP, because this could certify that associative phonagnosia is mainly due to a disconnection of the TVAs from structures of the voice extended system.
